# Examining the Effects of Supplemental Magnesium on Self-Reported Anxiety and Sleep Quality: A Systematic Review

**DOI:** 10.7759/cureus.59317

**Published:** 2024-04-29

**Authors:** Alexander Rawji, Morgan R Peltier, Kelly Mourtzanakis, Samreen Awan, Junaid Rana, Nitin J Pothen, Saba Afzal

**Affiliations:** 1 Department of Psychiatry and Behavioral Health, Ocean University Medical Center/Hackensack Meridian Health, Brick, USA; 2 Department of Psychiatry and Behavioral Health, Jersey Shore University Medical Center/Hackensack Meridian Health, Neptune, USA; 3 Department of Psychiatry and Behavioral Health, Hackensack Meridian School of Medicine, Nutley, USA

**Keywords:** ocd/anxiety disorders, mineral supplements, micro-nutrient, complementary and alternative medicine (cam), sleep quality & quantity, sleep, insomnia, clinical anxiety, magnesium oxide, magnesium supplements

## Abstract

Self-treatment with vitamin, mineral, and herbal supplements has become increasingly common among patients for the treatment of psychiatric disorders. Magnesium, in particular, is popular on social media for the treatment of anxiety and insomnia. Meanwhile, preclinical studies support associations between magnesium status, sleep quality, and symptoms of anxiety. The extent to which these claims are evidence-based is unclear. Therefore, a systematic review was performed to provide an updated examination of the clinical evidence on the use of magnesium for the treatment of the above conditions given the popularity of such supplements among patients and the public at large.

A thorough search of the PubMed database was performed and results were systematically reviewed using PRISMA guidelines. The search was limited to anxiety disorders and sleep disorders and included interventional trials only. Exclusion criteria included insufficient (<50 mg/12.5% of recommended daily allowance (RDA)) or unknown magnesium dose, >3 other potentially active compounds present in the formulation, and articles in languages other than English. This query returned 860 articles of which 15 met full inclusion criteria. Eight measured sleep-related outcomes, seven measured anxiety-related outcomes, and one examined both. Sleep quality was measured most frequently using the Pittsburg Sleep Quality Index (PSQI). Anxiety measures included self-reported measures such as the Hamilton Anxiety Scale. The majority of included studies demonstrated improvement in at least one sleep- or anxiety-related parameter. Five out of eight sleep-related studies reported improvements in sleep parameters, while two studies reported no improvements, and one reported mixed results. Five out of seven studies measuring anxiety-related outcomes reported improvements in self-reported anxiety.

Firm conclusions were limited by the heterogeneity of the data and the small number of participants involved in most of the studies. The dosages, formulations, and durations of the magnesium interventions used also differed across studies. Furthermore, some studies included additional, potentially active ingredients, further complicating interpretations. Given the generally positive results across studies, the preponderance of preclinical evidence, and minimal side effects, however, supplemental magnesium is likely useful in the treatment of mild anxiety and insomnia, particularly in those with low magnesium status at baseline. Notably, both negative anxiety trials featured populations with underlying endocrine factors likely contributing to their symptoms (patients with premenstrual symptoms and post-partum women). Nonetheless, larger, randomized clinical trials are needed to confirm efficacy and to establish the most effective forms and dosages of magnesium for the treatment of insomnia and anxiety disorders.

## Introduction and background

Interest in magnesium as a possible over-the-counter remedy for anxiety and sleep disorders is increasing among the lay public. On social media in particular, influencers frequently promote magnesium compounds and products such as anxiolytics and sleep aids. Magnesium-related videos uploaded to TikTok, a popular short-form video-sharing platform available on smartphones, commonly have hundreds of thousands to millions of views. The platform's impact on shaping perceptions and trends among its youthful audience is substantial, highlighting the importance of providing accurate and current information in the literature. Anecdotally, patients in our outpatient psychiatric practice increasingly inquire about magnesium-based products for the treatment of these disorders and may already be supplementing with the mineral upon presentation to our clinic. As such, there is a significant need for an up-to-date review of the evidence on these compounds as they relate to anxiety- and sleep-related symptoms as well as a review of their tolerability and safety. 

Magnesium is a ubiquitous element both in the earth's geology and human biology. It is the second most abundant cation in cells, with body stores of about 25 g [1]. It is known to be essential for the function of at least 300 or more enzymes in the body [2]. While frank deficiency is uncommon (and can result in seizures and arrhythmias among other severe adverse effects), it is thought that upward of half of the United States population does not meet the recommended daily allowance (RDA) for magnesium and is at risk for possible insufficiency [3]. The typical serum range for magnesium on lab assays is between roughly 0.75 and 0.95 mmol/L(~1.8 to 2.3 mg/dL) [4]. However, serum magnesium is believed to be an unreliable reflection of magnesium stores, as a significant portion of the body's magnesium is found in bone, muscle, and other tissues [1,3]. Other means of measuring magnesium status, including red blood cell magnesium content and urinary excretion of magnesium after magnesium oral challenge, are thought to possibly reflect body magnesium status more accurately [1]. 

Clinically, magnesium preparations have been used as prophylaxis in the treatment of migraine, arrhythmias, and acute asthma exacerbations, and have a long history of use in higher doses as a laxative [5]. Magnesium is also used in obstetrics for the treatment of eclampsia and preeclampsia and in neuroprotection for premature infants [5]. 

Importantly, magnesium exists in the supplement industry as a wide variety of compounds, both inorganic and organic forms, as various salts and amino acid chelates [2,6]. For instance, the mineral often exists on the formularies of hospitals as magnesium oxide (MgO) tablets or as a liquid laxative preparation as hydroxide or citrate (e.g., milk of magnesia). Various other forms of magnesium are also readily available commercially, including magnesium glycinate, taurate, sulfate, chloride, malate, threonate, aspartate, citrate, and orotate among others [6]. It is generally believed that organic forms, such as the amino acid chelates above, are absorbed and assimilated by the body more easily versus inorganic forms (e.g., MgO) [6]. 

Importantly, it has been discovered that magnesium ions function in the body as NMDA receptor antagonists [7,8]. Mg2+ ions occupy the NMDA receptor pore at typical neuronal membrane potential [7]. As neurons depolarize, the magnesium block is lifted allowing for the movement of Ca2+ and Na+ ions through the receptor pore [8]. Additionally, some research has shown magnesium may also exhibit some level of agonist activity at GABA-A receptors as well [7]. In a broader sense, magnesium often functions to oppose the excitatory action of calcium in the body, such as in the process of muscle contraction or within the central nervous system at NMDA receptors as described above [9]. 

Notably, animal models have repeatedly demonstrated the beneficial effects of magnesium administration in rodents as well as the adverse effects of induced magnesium depletion on anxiety and sleep parameters in rodents [10-15]. Regarding sleep, rodent models have shown magnesium depletion induces significant sleep disorganization, with decreased slow-wave sleep and increased wakefulness [14]. Similarly, multiple preclinical studies have demonstrated that magnesium depletion results in increased anxiety and depressive behaviors in rodents [16]. For example, in a study by Sartori et al. it was shown that magnesium-depleted rodents experienced an upregulated hypothalamic-pituitary-adrenal (HPA) axis, with significant increases in ACTH and CRH, which were then normalized with antidepressant treatment [17]. 

With the notable increase in interest in magnesium evident on social media platforms and among the general public, coupled with the consistent preclinical evidence, we aimed to systematically examine the existing evidence for the clinical application of magnesium supplements in the treatment of sleep and anxiety disorders in human subjects. This endeavor is intended to contribute valuable insights for future clinical practice. 

## Review

A systematic review of interventional trials was conducted using the PRISMA 2020 guidelines for new systematic reviews. The Pubmed database was queried up until the date the search was undertaken, 7/22/2023, using the search string: ((magnesium*[Title/Abstract]) OR (Mg*[Title/Abstract])) AND ((anxi*[Title/Abstract]) OR (GAD[Title/Abstract]) OR (Generalized Anxiety Disorder[Title/Abstract]) OR (insomnia[Title/Abstract]) OR (sleep*[Title/Abstract]) OR (panic*[Title/Abstract])).

This search captured various derivatives of the word “anxiety” by the included ‘anxi*’ term and in general was designed to capture a range of anxiety disorders. It was also designed to capture general sleep disorders with the sleep* term, particularly studies relating to insomnia. This search was designed to exclude stress or stress-related disorders, even though magnesium is often promoted as a stress-reducing compound anecdotally and on social media. 

Only interventional studies were included in the review (either randomized control trial (RCT) or observational). Inclusion criteria required sufficient reporting detail and inclusion of all relevant variables including magnesium dose, form, and period of administration. Inclusion was not restricted by the age of the participant or the form of magnesium. Included studies featured standardized instruments to measure anxiety and sleep outcomes (or polysomnography in the case of sleep disorders). Only human studies were included (all animal studies were excluded). 

Exclusion criteria included an insufficient dose of magnesium, defined as ≤50 mg/12.5% of RDA for purposes of this review, as such amounts were considered unlikely to be therapeutic. Additional exclusion criteria included unknown magnesium dose, the presence of >3 other potentially active compounds present in the formulation, and articles in languages other than English. The limitation on >3 potentially active ingredients was included to minimize the difficulty in attributing positive effects to magnesium that would result from the inclusion of multiple other active ingredients (Table 1). 

**Table 1:** Inclusion and exclusion criteria RCT, randomized control trial

Inclusion criteria
· Interventional studies only (either RCT or observational)
· Sufficient reporting detail of all relevant variables including magnesium dose, form, and time length of administration
· Standardized instruments to measure anxiety or sleep outcomes (or polysomnography in the case of sleep disorders)
· Human studies only (all animal studies were excluded)
· Inclusion not restricted by age of participant or type of magnesium salt
Exclusion criteria
· Insufficient dose of magnesium, defined as ≤50 mg/12.5% of RDA for purposes of this review or unknown magnesium dose
· Presence of >3 other potentially active compounds in the formulation
· Manuscript language other than English

Following completion of the formal search and exclusion of articles as described in the PRISMA flow chart, article information was extracted including the following variables: study name, authors, year, country, study design, number of participants and specific population studied, inclusion/exclusion criteria of the study, Mg form, dose, presence of additional active ingredients, duration, outcome variables, and description of results. 

Results and discussion 

**Figure 1:**
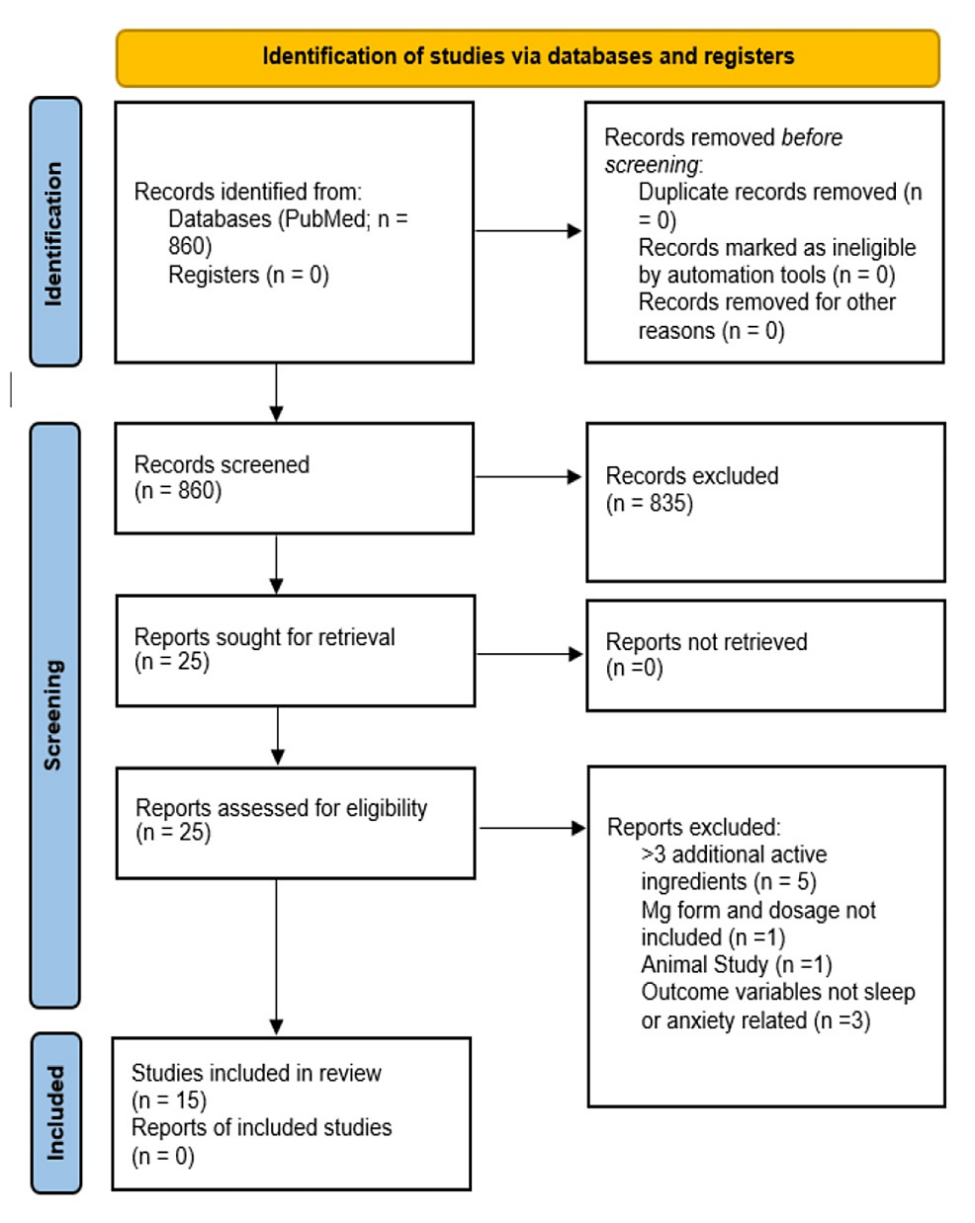
PRISMA flow diagram for systematic reviews

The search revealed a total of 860 articles, of which 835 were excluded using the above criteria after reviewing the title/abstract. The full texts of the remaining articles were obtained from the Jersey Shore University Medical Center (JSUMC) medical library and reviewed against the above criteria. Of these, an additional 10 articles were excluded following full-text review for the following reasons: Five featured >3 potentially active ingredients, one lacked sufficient information detailing the intervention (lack of Mg dose, form, and duration of administration), one was an animal study, and three did not include sleep- or anxiety-related outcome variables. The full PRISMA flowchart is in Figure 1.

Of the 15 studies included in the final systematic review, eight studies reported sleep-related outcomes and seven reported anxiety-related outcomes. Nearly all studies were conducted in the Middle East or Europe, with only one study conducted in the USA. The two most common countries of origin were France (four studies, one reporting sleep outcomes and three reporting anxiety outcomes) and Iran (five studies, three reporting sleep outcomes and two reporting anxiety outcomes). All but four studies were RCTs. The vast majority of studies used MgO as the specific form of magnesium studied, with the maximum daily dose used in any study being 729 mg of magnesium daily. Adverse events were generally mild and uncommon, with the most commonly reported adverse effect being loose stool. 

Sleep-related studies 

Eight of the 15 studies included in the review reported on magnesium’s effect on sleep quality. The primary sleep metric used by the majority of studies was the Pittsburgh Sleep Quality Index (PSQI), a self-reported questionnaire validated in clinical populations including patients with fibromyalgia, psychiatric disorders such as major depressive disorder, and patients with sleep disorders such as insomnia [18]. The PSQI consists of 19 self-reported items across seven subcategories, including sleep quality, latency, total sleep time, disturbances of sleep, and dysfunction during the day. Six of the eight sleep-related studies used the PSQI to measure subjective sleep. Four out of the six of these studies featured positive results with improvements in the PSQI. Overall, five out of eight sleep-related studies reported generally positive results on sleep parameters. Two studies reported negative results and one featured mixed results. Six out of eight were RCT study designs, while two were open-label pilot studies. 

Five studies used MgO as the intervention, ranging in dosage from 250 mg to 729 mg. Another used magnesium chloride, dosed at 100 mg of a specially formulated, slow-release delivery system. One study used magnesium citrate and one used magnesium L-aspartate. The length of interventions ranged considerably from five days to 10 weeks. Four out of the five studies using MgO reported generally positive results either by self-assessment via PSQI or by EEG measurements. Notably the one negative study featuring MgO as the intervention also used the lowest dose of MgO out of all the studies, 250 mg. Furthermore, the dose was administered early in the day, given with breakfast, where it was likely metabolized and excreted by bedtime. The study using MgCl also reported no significant improvements in sleep-related outcomes. This study, featuring adults with fibromyalgia, dosed MgCl at 100 mg of elemental magnesium, which was the lowest dose of Mg across all sleep-related studies. The investigators used a formulation that reportedly featured higher absorption to counter the lower dose of Mg, though there were no changes in serum or RBC magnesium by the end of the study, in addition to no change in sleep quality as measured by PSQI. 

The study by Hornyak et al. (2004) using magnesium L-aspartate featured among the highest daily dose of Mg of any sleep-related study included in the review (729 mg). This study, conducted in primary alcohol-dependent patients during subacute withdrawal from alcohol, described improvements in both subjective sleep quality as measured by PSQI as well as objective EEG measures such as significantly decreased sleep onset latency, from an average of 40.6 minutes pre-treatment to 21.7 minutes (P=0.03). Another study featuring 729 mg of magnesium (as MgO) by Held et al. conducted in a population of healthy elderly subjects aged 61-81 years demonstrated an increase in slow wave sleep by EEG from 10.1 minutes to 16.5 minutes, P< 0.05, along with increases in delta power (a measure of slow wave sleep) and sigma power. 

The only study featuring magnesium citrate (Nielsen et al., 2010), conducted in a population of adults >51 years with baseline PSQI >5 (reflecting relatively poor baseline sleep quality), demonstrated significant improvements in sleep quality regardless of allocation to placebo or treatment group. PSQI declined overall from 10.4 to 6.6 (P<0.0001) across both groups. Curiously, RBC magnesium increased significantly in both the treatment group as well as the placebo group, suggesting a possible change in behavior by the participants due to being observed (i.e., the Hawthorne effect). A subset of patients in this study with high baseline inflammatory markers (as demonstrated by high sensitivity CRP >3.0) did demonstrate a decrease in hsCRP with magnesium administration (Table 2). 

**Table 2:** Summaries of magnesium studies reporting sleep-related outcomes RCT, randomized control trial; PSQI, Pittsburg Sleep Quality Index; PLMS, Periodic Limb Movements in Sleep; RLS, restless leg syndrome; DASS, Depression, Anxiety, Stress Scales; RDA, recommended daily allowance; DHEA, dehydroepiandrosterone; SWS, slow wave sleep

Authors	Country	Study design	Participants		Inclusion/exclusion criteria		Intervention					Outcome measures	Relevant results	Positive/negative
			N	Population	Inclusion	Exclusion	Magnesium form	Dose	Additional therapies	Duration	Description			
Gholizadeh‐Moghaddam et al. (2022) [19]	Iran	Double‑blind RCT	64; control group N=32, tx group N=32	Females aged 18-45	Females sex; age 18-45; PCOS dx per Rotterdam criteria	New or change in medication within two weeks; peri- or post-menopausal status; concurrent use of any other vitamin or mineral supplement; pregnancy	MgO	250 mg	No	10 weeks	250 mg MgO taken for 10 weeks after breakfast	Sleep quality as assessed by the PSQI; serum concentration of magnesium, DHEAs, and testosterone; hirsutism as assessed by the Ferriman–Gallwey questionnaire	Sleep quality did not significantly improve after 10 weeks in either the treatment or the control group; no significance between group differences in regard to sleep quality	N
Saba et al. (2022) [20]	Iran	Single-blind controlled trial	60; control group N=30, tx group N=30	Hospitalized adults undergoing open heart surgery	Age <70 years old; candidate for elective CABG surgery	Hx of atrial fibrillation prior to CABG surgery; hx of liver or renal failure; hx of stroke or recent TIA; postoperative respiratory failure; liver or kidney failure; emergency surgery during the study period; chronic diarrhea; allergy to study drug; hx of sleep disorders; hx of anxiety or depression; parenteral MgSO4 tx	MgO	500 mg	No	Five days	500 mg MgO for five days (two pills of 250 mg MgO each)	Sleep quality as assessed by the PSQI	The mean PSQI score was significantly lower in the treatment group versus the control group at the end of the study period; within-group and between-group differences in PSQI score were significant (P=0.001 and P=0.021, respectively)	P
Nielsen et al. (2010) [21]	USA	Double‑blind RCT	96; control group N=47, tx group N=49	Adults aged >51 years with PSQI >5	Age >51 years; PSQI >5; normal CBC, liver and kidney function tests	Use of supplements containing >100 mg of magnesium; BMI >40; COPD; use of O_2_ or CPAP; use of ACE-inhibitors; use of magnesium or potassium retaining drugs	Magnesium citrate	320 mg	No	Eight weeks	320 mg of magnesium citrate across five capsules - two taken with AM meal, one taken with noon meal, and two taken with PM meal	Sleep quality as assessed by the PSQI	PSQI declined significantly from 10.4 to 6.6 (P<0.0001); RBC magnesium increased significantly in both the treatment and placebo groups; hsCRP decreased significantly in the subset of pts with hsCRP>3.0 with magnesium treatment but not placebo	Mixed
Hornyak et al. (1998) [22]	Germany	Open pilot study	10	Patients with RLS or PLMS	Diagnosed RLS or PLMS	Severe RLS or PLMS; abnormal pre-study medical workup (ECG, EEG, CBC); elevated creatinine; use of sedatives or psychiatric medication within four weeks; uremia; chronic bronchitis; Fe deficiency; pregnancy	MgO	291.6 mg	No	Four to six weeks (average 5.1 weeks)	291.6 mg MgO QHS	Sleep quality as assessed by the PSQI; sleep EEG; subjective sleep quality measured by SF-A questionnaire; # of periodic limb movements during sleep	PLMS-associated w/ arousals decreased significantly from 17±7 versus 7±7 events per hour of total sleep time (P<0.05); sleep efficiency increased from 75±12% to 85±8% (P<0.01); PLMS w/o arousals trended lower from 33±16 to 21±23 (P=0.07)	P
Hornyak et al. (2004) [23]	Germany	Open pilot study	11	EtOH-dependent patients in subacute withdrawal	Primary EtOH dependence in subacute withdrawal (two weeks since last drink)	Cognitive impairment; major medical comorbidities (e.g., renal failure, heart failure); OSA, tx with anti-craving medications; magnesium supplementation within two weeks; prolonged EtOH withdrawal; depression; use of psychoactive medication within seven days	Magnesium L-aspartate	729 mg	No	Four weeks	243 mg of magnesium aspartate q AM, 486 mg of magnesium Aspartate QHS	Sleep quality measured by PSQI; sleep EEG; subjective sleep quality measured by SF-A; # of periodic limb movements during sleep	Sleep-onset latency decreased significantly from 40.6 to 21.7 minutes (P=0.03); PSQI subjective sleep quality score improved significantly from a mean of 8.1 to 5.8 (P=0.05); total sleep time and slow wave sleep time increased but did not achieve significance	P
Held et al. (2002) [24]	Germany	Double-blind, randomized, placebo-controlled crossover trial	12; control group N=6, tx group N=6	Healthy elderly subjects aged 61-81	Healthy status, as measured by bloodwork, EEG, and ECG	Personal or family psychiatric hx; personal or family hx of neurocognitive disorder; hx of substance abuse; hx of transmeridian flight within three months; shift work; sleep‑related movement disorders or respiratory disorders	MgO	243 mg for three days, 486 mg for three days, 729 mg for 14 days	No	20 days	20 days of active tx - 243 mg for three days (dosed in AM), 486 mg for three days (243 mg dosed in AM and noon), 729 mg for 14 days (243 mg dosed in AM, noon and PM)	Sleep EEG; serum ACTH, cortisol, AVP, renin, ATII, aldosterone, magnesium	Significant increase in SWS from 10.1 min to 16.5 min (P<0.05); increase in delta power and sigma power (P<0.05); renin and aldosterone increased and cortisol decreased significantly versus the control (P<0.05)	P
Macian et al. (2022) [25]	France	Double‑blind RCT	76; control group N=38, tx group N=38	Adults with fibromyalgia experiencing moderate to severe stress	Aged >18 years; dx of fibromyalgia (as per ACR 2016 criteria); >18 on the DASS stress subscale	Pregnancy; breastfeeding; women of childbearing age not using contraceptive; diabetes mellitus; kidney disease (CrCl <30); hypermagnesemia (plasma magnesium >1.05 mmol/L); antibiotic treatment; other magnesium-containing medications/supplements	MgCl	100 mg	No	Four weeks	100 mg daily of low-dose continuous-release MgCl taken for four weeks	Change in sleep quality as assessed by the PSQI; serum and RBC magnesium measurements	No significant change in sleep quality as measured by PSQI after four weeks; no significant change in serum and RBC magnesium after four weeks	N
Abbasi et al. (2012) [26]	Iran	Double‑blind RCT	46; control group N=23, tx group N=23	Adults aged 60-75; average age 65±4.6 years	Insomnia as dx by ISI; BMI 25-34.9; magnesium intake <75% of RDA; serum magnesium <0.95 mmL	Tx with loop diuretics, cyclosporine, digoxin, amphotericin, any hormonal therapy; renal disease; acute heart failure; sleep‑related movement disorders or respiratory disorders; any psychiatric disorder; recent stressful life event (e.g., divorce; death or acute illness of a family member); substance/alcohol abuse; transmeridian flight within six weeks	MgO	500 mg	No	Eight weeks	500 mg MgO for eight weeks	Sleep time, sleep efficiency, ISI score, sleep onset latency; serum cortisol, renin, and melatonin	Increased sleep time (P=0.002) and sleep efficiency (P=0.03); increased concentration of serum renin (P<0.001) and melatonin (P=0.007); decrease of ISI score (P=0.006), sleep-onset latency (P=0.02), and serum cortisol concentration (P=0.008); early morning awakening (P=0.08), serum magnesium concentration (P=0.06), and total sleep time (P=0.37) did not did not achieve significance	P

Anxiety-related studies 

Seven of the 15 studies included in the systematic review investigated the effects of magnesium supplementation on measures of anxiety. The exact measures used varied across studies with the most common measure being the Hamilton Anxiety Rating Scale (HAM-A), though this was only used in two of the studies [27]. The HAM-A is a commonly used, 14-question assessment of anxiety, with each item rated on a zero to four scale corresponding with "not present" to "very severe." Other anxiety outcome measures used included the Revised Child Anxiety and Depression Scale (RCADS), the Spielberger State-Trait Anxiety Inventory (STAI), the Depression Anxiety Stress Scales (DASS), and the Hospital Anxiety and Depression Scale (HADS) [28-31]. Specific populations studied varied considerably as well and included pediatric migraine patients, women suffering anxiety-related premenstrual symptoms, postpartum women, adults experiencing moderate to severe stress, and hospitalized adults undergoing open heart surgery, as well as patients with generalized anxiety disorder and adjustment disorder with anxiety. Five out of seven studies were RCT study designs while two studies were prospective cohort studies. Intervention length varied significantly from five days to six months, though most trials were between four and eight weeks. Five of the studies used MgO, one used magnesium sulfate, and one used magnesium lactate dihydrate. Importantly, several of the studies included magnesium combined with (three or fewer) other potentially active ingredients in the treatment arms. Three studies included magnesium with vitamin B6 (at doses of 1.4 mg, 30 mg, and 50 mg daily, respectively). Another study used two plant extracts - *Crataegus oxyacantha *and *Eschscholtzia californica* - along with 150 mg of elemental magnesium in the form of MgO as the study intervention. Finally, another study combined magnesium and vitamin B6 with 200 mg of fish protein hydrolysate. 

Overall, five out of seven studies featuring anxiety-related outcomes reported positive results. The two trials with the greatest reductions in anxiety scores (Noah et al., 2020; Oddoux et al., 2022) used relatively high doses of magnesium (300 mg elemental magnesium each) complexed with other active ingredients (30 mg of pyridoxine and 1.4 mg of pyridoxine plus 200 mg of fish protein hydrolysate). The one study with clear negative results (Edalati Fard et al., 2017) used 320 mg of magnesium sulfate that contained 64.6 mg of elemental magnesium - the lowest amount of magnesium used in any of the 15 studies included in our review. Another study (De Souza et al., 2000) reported decreased self-reported anxiety scores only when magnesium was combined with 50 mg of pyridoxine. However, urinary excretion of magnesium during this trial did not significantly change, leading the authors to question the absorption of the MgO in the trial (Table 3). 

**Table 3:** Summaries of magnesium studies reporting anxiety-related outcomes GAD, generalized anxiety disorder; MHQ, menstrual health questionnaire; HAM-A, Hamilton Anxiety Rating Scale; DASS, Depression, Anxiety, Stress Scales; OCPs, oral contraceptive pills; RCADS, Revised Child Anxiety and Depression Scale; HADS, Hospital Anxiety and Depression Scale; CGI, clinical global impression; STAI, Spielberger State-Trait Anxiety Inventory

Authors	Country	Study design	Participants		Inclusion/exclusion criteria		Intervention					Outcome measures	Relevant results	Positive/negative
			N	Population	Inclusion	Exclusion	Magnesium form	Dose	Additional therapies	Duration	Description			
Saba et al. (2022) [20]	Iran	Single-blind controlled trial	60; control group N=30, tx group N=30	Hospitalized adults undergoing open heart surgery	Age <70 years old; candidate for elective CABG surgery	Hx of atrial fibrillation prior to CABG surgery; hx of liver or renal failure; hx of stroke or recent TIA; postoperative respiratory failure; liver or kidney failure; emergency surgery during the study period; chronic diarrhea; allergy to study drug; hx of sleep disorders; hx of anxiety or depression; parenteral MgSO4 tx	MgO	500 mg	No	Five days	500 mg MgO for five days (two pills of 250 mg MgO each)	Anxiety and depressive sx as assessed by the HADS	The mean HADS score was significantly lower in the treatment group versus the control group (P=0.007)	P
Kovacevic et al. (2017) [32]	Serbia	Prospective cohort study	32	Pediatric migraine patients without comorbidities	Ages 7-17; dx of migraine at least one month prior; no previous migraine prophylaxis	Presence of any other medical, neurological, or psychiatric disorder	MgO; magnesium glycinate	4-6 mg/kg/day	NSAIDs for acute migraine attacks during the study period	Six months	MgO or magnesium glycinate, dosed at 4-6 mg/kg/day for six months	Anxiety and depressive sx as assessed by the RCADS	Self-reported anxiety scores decreased significantly between baseline and six months (P=0.001); scores between baseline and three months trended lower but did not achieve significance (P=0.115)	P
De Souza et al. (2000) [33]	United Kingdom	Double-blind randomized controlled crossover trial	44 (crossover trial; each served as own control)	Women with premenstrual anxiety sx	Premenstrual scores >30% higher than post-menstrual sx as measured by the MHQ	Patients already taking any vitamin or mineral supplement; any medication aside from OCPs	MgO	200 mg	Pyridoxine 50 mg	Five menstrual cycles	Crossover to alternate treatment during each subsequent menstrual period (1) MgO 200 mg, (2) MgO + pyridoxine 50 mg, (3) pyridoxine 50 mg, (4) placebo	Self-reported, subjective anxiety sx reported on a five-point ordinal scale; 24-hour magnesium and creatinine output	Significantly lower anxiety scores during treatment with a combination of magnesium and B6 compared with other treatments (P=0.04); urinary magnesium did not differ significantly between groups	Mixed
Oddoux et al. (2022) [34]	France	Prospective cohort study	93	Adults aged 18-70 with adjustment disorder with anxiety, at least mild-moderate in severity	Adults aged 18-70 years; HAM-A score >20	Previous treatment for anxiety within the prior three months (including psychotherapy); the presence of any other mental illness; anxiety lasting >3 months; history of substance abuse; >10 mg/kg of caffeine/day; >1 pack of cigarettes/day; pregnancy; breastfeeding; severe medical comorbidity	MgO and magnesium bisglycinate	300 mg (270 mg as MgO and 30 mg as magnesium bisglycinate)	200 mg of fish protein hydrolysate ("Gabolysat®") and 1.4 mg of vitamin B6 (as pyridoxal chlorate)	Four weeks	Four weeks of 300 mg magnesium, 200 mg fish protein hydrolysate, and 1.4 mg B6 combo, taken with breakfast	% of patients with ≥50% decrease in HAM-A score; change in HAM-A score; change in Clinical Global Index Scale	41.9% (39/93) experienced a ≥50% decrease in HAM-A score (primary endpoint); mean HAM-A score decreased by 12.1 +/- 5.7 points (P<0.001); 75.3% improved significantly or very significantly on CGI scale	P
Fard et al. (2017) [35]	Iran	Triple-blind RCT	99; control group N=33, each tx group N=33	Postpartum women having given birth within 48 hours	≥18 years old; living in Tabriz, Iran; low-risk pregnancy	Depression (12 or greater on the Edinburgh Scale); any psychiatric history; complications with childbirth; NICU admission or death in the infant; significant stressful event (divorce, hospitalization, or death of relative); chronic illness; liver or kidney disease; hx of infertility or previous miscarriage	MgSO4	320 mg (64.6 elemental magnesium)	None	Eight weeks	Eight weeks of either placebo, 27 mg of ZnSO_4_, or 320 mg of MgSO_4_	STAI; Edinburgh Postnatal Depression Scale	No significant differences were found in depression and anxiety scores between the placebo, zinc, and magnesium groups	N
Noah et al. (2020) [36]	France	Single-blind RCT	264 control group N=132, tx group N=132	Adults aged 18-50 experiencing moderate-severe stress	Ages 18-50 years old; moderate to severe stress as measured by >18 on the DASS stress subscale; low magnesium status as defined by serum values of 0.66-0.84 mmol/L	Use of levodopa, quinidine, and proton-pump inhibitors within the three months prior to screening; alcohol intake of >3 drinks per day; hx of substance abuse; type 1 and type 2 diabetes mellitus; moderate or severe kidney disease; severe hypomagnesemia (serum magnesium of <0.45 mmol/L)	Magnesium lactate dihydrate	300 mg elemental magnesium total (across six tablets)	30 mg of pyridoxine (in tx group; just magnesium lactate alone as control)	Eight weeks	Eight weeks of either magnesium lactate 100 mg AM, 100 mg at noon, and 100 mg PM (control group) or eight weeks of magnesium lactate-B6 100 mg-10 mg AM, 100 mg-10 mg at noon, and 100 mg-10 mg PM (tx group)	DASS depression and anxiety subscales	DASS anxiety subscale scores decreased significantly from baseline in both the magnesium and magnesium+B6 groups (P<0.05); no significant difference between groups was found	P
Hanus et al. (2003) [37]	France	Double-blind RCT	264 control group N=134, tx group N=130	Patients with mild-moderate GAD	Adults >18 years old with mild-moderate GAD	Other axis 1 disorders; treatment with psychotropic medication, sedatives, or magnesium within one month of study	MgO	248.7 mg MgO (150 mg elemental magnesium)	Extracts of the plants: *Crataegus oxyacantha* and *Eschscholtzia californica*	90 days	Two tablets of 124.35 mg MgO and plant extracts of *Crataegus oxyacantha* and *Eschscholtzia californica* (75 mg elemental magnesium per tablet) before AM and PM meal. 300 mg MgO total daily	Anxiety as assessed by the HAM-A; Patient self-reported anxiety scores (VAS 0-100 scale, 100=very anxious); % responsive (>50% reduction in sx); physician’s CGI	Total HAM-A score decreased significantly more in the tx group versus placebo (-10.6±1.2 versus -8.9±1.2, P=0.005); VAS subjective anxiety score decreased significantly more in tx group versus placebo (-38.5 versus -29.2, P=0.005); significantly greater response rate in tx group versus placebo for both the HAM-A and the VAS scores: 45% versus 32%, P=0.017 and 58% versus 43%, P=0.008, respectively	P

The two studies in which magnesium was not effective in reducing anxiety scores also featured the two populations with possible significant hormonal perturbations contributing to their symptoms, premenstrual and postpartum women. It remains possible that magnesium is less effective in these populations for the treatment of anxiety. 

## Conclusions

This systematic review was undertaken to identify and summarize available literature on the use of magnesium in the treatment of anxiety and sleep disorders given the increasing use of the mineral by the lay public for the treatment of these conditions. Overall, most studies were small with significant heterogeneity present among the dosages and treatment periods used and the populations studied, thus making firm conclusions and generalizability of findings difficult. However, higher doses of magnesium appear to be more effective in addressing anxiety and sleep disturbances, as all studies with negative results used low comparative dosages of the mineral in their treatment interventions. Additionally, magnesium may be more effective in combination with other active ingredients, specifically vitamin B6, in the treatment of anxiety disorders. It should be noted that vitamin B6 is heavily involved in neurotransmitter synthesis, which may underlie this possible trend. A determination of the optimal form of magnesium for use in anxiety and sleep disorders was not possible in the present review as the vast majority of studies used MgO. As described previously, inorganic forms of magnesium (e.g., MgO) have been suggested to be less readily absorbed in the gastrointestinal tract. Furthermore, larger studies may likely favor other forms of magnesium over the oxide form in the treatment of these disorders in the future. Adverse events in the included studies were mild if present - most commonly increased bowel movements, a well-known side effect of magnesium administration. Given the mineral’s use as a laxative in high doses, it is likely this adverse effect is dose-dependent. 

In general, despite notable heterogeneity, the majority of included trials demonstrated at least modest positive results with regard to sleep quality and anxiety across diverse populations. These findings are consistent with animal-based evidence as well as magnesium’s known receptor activity in the central nervous system. Larger, well-designed trials are needed to further characterize specific forms and doses for routine use of magnesium in clinical practice. 
